# Retention index of FDG-PET/CT SUVmax of the primary tumor in non-small cell lung cancer as a predictor of lymph node metastasis: a retrospective study

**DOI:** 10.1186/s41824-022-00141-6

**Published:** 2022-09-27

**Authors:** Toshinari Ema, Hideaki Kojima, Shinji Mizuno, Tatsuo Hirai, Mikako Oka, Hiroshi Neyatani, Kazuhito Funai, Norihiko Shiiya

**Affiliations:** 1 Department of Thoracic Surgery, Fujieda Municipal General Hospital, 4-1-11 Surugadai, Fujieda City, Shizuoka 426-8677 Japan; 2 Department of Radiology, Heisei Memorial Medical Center, Shizuoka, Japan; 3 First Department of Surgery, Hamamatsu University School of Medicine, Shizuoka, Japan

**Keywords:** Non-small cell lung cancer, Lymphatic metastasis, Positron-emission tomography/computed tomography, 18F-FDG

## Abstract

**Background:**

Accurate staging of non-small cell lung cancer is key in treatment planning and prediction of prognosis. We investigated the correlation between the maximum standardized uptake value (SUVmax) retention index (RI) of the primary tumor and lymph node metastasis in non-small cell lung carcinoma. We also evaluated the tendencies according to the histological types.

**Methods:**

We retrospectively evaluated 218 non-small cell lung cancer (NSCLC) tumors from 217 patients who underwent preoperative fluorodeoxyglucose-positron emission tomography/computed tomography (PET/CT) followed by lung surgery and lymph node resection between July 2015 and August 2020. All primary tumors were calculated as the SUVmax at 50 min (SUVmax_early_ [SUVmax_e_]) and 120 min (SUVmax_delayed_ [SUVmax_d_]), and RI. The clinicopathological factors of interest were compared based on lymph node metastasis status and NSCLC histopathological subtype.

**Results:**

The median SUVmax_e_ and SUVmax_d_ of the primary tumors were 3.3 and 4.2, respectively, and the median RI was 0.25. The RI was significantly higher in the pN(+) (*n* = 44) group (0.30) compared to the pN0 (*n* = 174) group (0.24) (*p* = 0.01). In patients with adenocarcinoma (*n* = 145), the RI was also significantly higher in the pN(+) (*n* = 29) group (0.29) compared to the pN0 (*n* = 116) group (0.16) (*p* < 0.01). A high RI of the primary tumor was an independent risk factor for lymph node metastasis, particularly in patients with adenocarcinoma (odds ratio: 12.30, *p* < 0.05).

**Conclusions:**

The RI of primary NSCLC tumors can help predict lymph node metastases, particularly in patients with adenocarcinoma.

## Background

Accurate staging of non-small cell lung cancer (NSCLC), especially the preoperative diagnosis of lymph node metastasis, plays a key role in planning treatment and guiding prognostication in affected patients (Takahashi et al. 2019). Lymph node metastasis can be ascertained using invasive or noninvasive methods. Positron emission tomography/computed tomography (PET/CT) using 18F-fluorodeoxyglucose (18F-FDG), a glucose analog shown to be useful for detecting malignancy, is a noninvasive method widely used to help stage NSCLC. The maximum standardized uptake value (SUVmax) of the primary tumor is risk factor for nodal metastasis, and the typical SUVmax cutoff value is 2.5–4.0 (Takahashi et al. 2019; Noda et al. 2016; Nakamura et al. 2015; Karam et al. 2018). Dual-time-PET/CT is also widely implemented, with scanning being performed for almost 1–2 h following injection to distinguish between malignancy and inflammation (Shinya et al. 2009). The effectiveness of the dual-time-point (DTP) PET/CT retention index (RI) as a predictor of lymph node metastasis has been described (Shinya et al. 2009, 2019; Kim et al. 2012). However, these studies reported that the SUV RI of the lymph node itself was effective for predicting lymph node metastasis. Further, no studies have examined the role of the primary tumor’s SUVmax RI in the prediction of lymph node metastasis. In a real-world clinical setting, the SUVmax of hilar and mediastinal lymph nodes is often not calculated. In this study, we therefore explored the meaning of the primary tumor’s RI as a predictor for lymph node metastasis in a real-world healthcare setting. This study investigated the correlations between the SUVmax RI of the primary tumor and lymph node metastasis in different histological subtypes of NSCLC.

## Methods

### Patients

In this observational study, we retrospectively evaluated 218 NSCLC tumors from 217 enrolled patients who underwent a preoperative FDG-PET evaluation and subsequent surgical resection (lobectomy, bilobectomy, pneumonectomy, or segmentectomy) and lymphadenectomy of ND1a-ND2a-2 between July 2015 and August 2020.

One of the male patients had two evaluable tumors, although the regional lymph node of each tumor was separated. We enrolled consecutive operative cases in that period that were clinically diagnosed as resectable lung cancer with clinical staging cN0–2. We excluded patients who underwent wedge resection with no lymph node dissection and those who received neoadjuvant chemotherapy. Among the cases with adenocarcinoma, we excluded patients with adenocarcinoma in situ (AIS), minimally invasive adenocarcinoma (MIA), and former bronchioloalveolar carcinoma (BAC). Tumor size, lymph node metastasis, and histological types were all determined using surgically resected specimens. Pathological staging of all the patients was performed according to the 7th edition of the International Union Against Cancer and the American Joint Committee on Cancer TNM staging system for lung cancer (Rusch et al. 2009). The SUVmax was calculated for the primary tumor only. The study protocol was reviewed and approved by the institutional review board.

### Nuclear imaging and analysis

Patients were asked to fast for at least 5 h before the examination, after which blood glucose levels were determined. The patients then received an intravenous injection of 185 MBq/body (at the time of inspection) of 18F-FDG and rested for approximately 50 min before scanning. PET/CT was performed for all the patients at Heisei-Memorial Medical Center (Fujieda, Japan) using a multidetector CT integrated high-resolution PET/CT scanner (Aquiduo PCA-7000B system, Canon Medical Systems Corporation, Otawara, Tochigi, Japan). Image acquisition started 50 min after FDG (early-phase) injection and 2 h after the injection (delayed-phase) in a relaxed supine position.

An unenhanced CT scan was performed first, from the inguinal region of the thigh to the head, with the following settings: 120 kV, 80 mA (average, max 110 mA), helical pitch 15, and section thickness 4 mm, which matched the PET section thickness. A PET scan was performed that covered the identical transverse field of view immediately after the CT scan. The acquisition time for the PET scan was 2 min. Patients were in normal shallow respiration during image acquisition. The PET datasets were reconstructed iteratively using the CT data for attenuation correction, and co-registered images were displayed on a workstation (Aquiduo Vox-Base Managaser 2.8, J-MAC System).

Two experienced nuclear physicians blinded to the histologic results interpreted all 18F-FDG-PET/CT findings. For the semiquantitative analysis of FDG uptakes, the SUV was adopted. The SUVs were calculated using lean body mass according to the following formula:

$${\text{SUV}} = \frac{{{\text{radioactivity}}\,{\text{in}}\,{\text{regions}}\,{\text{of}}\,{\text{interest}}\,\left( {{\text{ROI}}} \right)\,\left( {\text{MBq/mL}} \right){ } \times {\text{body}}\,{\text{weight}}\,\left( {\text{k}} \right)}}{{{\text{injected}}\,{\text{FDG}}\,{\text{radioactivity}}\,\left( {{\text{MBq}}} \right)}}$$

Circular regions of interest were drawn to encompass primary lung tumor contours on the attenuation corrected PET/CT images.

The SUVmax RI was calculated using the following formula:

$${\text{RI}} = \frac{{{\text{SUVmax}}\,{\text{delayed}}\,{\text{point}} - {\text{SUVmax}}\,{\text{early}}\,{\text{point}}}}{{{\text{SUVmax }}\,{\text{early}}\,{\text{point}}}}$$

The SUVmax and RI were calculated for the primary tumor only.

### Statistical analyses

Statistical analyses were performed using the EZR software package (version 1.40; Saitama Medical Center, Jichi Medical University, Saitama, Japan) (Kanda 2013), a graphical user interface for R (The R Foundation for Statistical Computing, Vienna, Austria). The EZR software is a modified version of the R commander designed to facilitate the addition of statistical functions frequently used in biostatistics. Categorical data were presented as frequencies and compared between patient groups using Fisher’s exact test. Data which fit normal distribution assumptions were described as the mean ± standard deviation. Non-normal data were described as the median and interquartile range. Parametric and nonparametric data were analyzed using an unpaired Student’s *t* test and the Mann–Whitney U test, respectively. The receiver operating characteristic (ROC) curve was used to analyze diagnostic efficacy, sensitivity, and specificity for NSCLC lymph node metastasis, and the cutoff point was calculated using the Youden index. The linear correlations between the pathological T size and RI were calculated using Pearson’s correlation coefficients. Logistic regression analyses were performed to identify independent predictors of risk for pathological lymph node metastasis. All *p* values were nominal, and a two-sided *p* value < 0.05 was considered statistically significant.

## Results

### Patient characteristics

In total, data relevant to 218 NSCLC tumors from 217 patients were retrospectively reviewed. The patient characteristics are summarized in Table 1. This study included 139 men and 78 women (mean age, 69.6 ± 9.5 years). One of the male patients had two evaluable tumors, and the regional lymph node of each tumor was separated. In all cases, a preoperative clinical stage of N0–N2 was considered indicative of curative resection. We diagnosed clinical lymph node metastasis when the short axis of the node was larger than 1 cm on CT, or when the lymph node had high FDG accumulation.

**Table 1:** Characteristics of the patients and tumors included in this study

Characteristics	N (%)
Sex	
Male	139 (64.0%)
Female	78 (35.9%)
Lobe	
RU	67 (30.7%)
RM	13 (6.0%)
RL	51 (23.4%)
LU	41 (18.8%)
LL	46 (21.1%)
Histological type	
Adenocarcinoma	145 (66.5%)
Squamous	53 (24.3%)
Other	20 (9.2%)
cN	
0	187 (85.8%)
1	26 (11.9%)
2	5 (2.3%)
pN	
0	174 (79.8%)
1	27 (12.4%)
2	17 (7.8%)
pN	
0	174 (79.8%)
1 or 2	44 (20.2%)
Age, years	69.6 ± 9.5
SUVmax_e_	3.3 [1.73, 6.58]
SUVmax_d_	4.2 [2.0, 8.68]
RI	0.25 [0.08, 0.37]
pT (mm)	22.0 [15.8, 30.0]

*n* = 218 for all characteristics, except sex (*n* = 217). Data are presented as n (%), mean ± standard deviations, or median [interquartile ranges]

*pN* pathological lymph node metastasis, *RU* right upper lung lobe, *RL* right lower lung lobe, *RM* right middle lung lobe, *LU* left upper lung lobe, *LL* left lower lung lobe, *SUVmax*_*e*_ early maximum standardized uptake value, *SUVmax*_*d*_ delayed maximum standardized uptake value, *RI* retention index, *pT* pathological tumor size

All lesions were surgically resected via lobectomy, segmentectomy, bilobectomy, or pneumonectomy accompanied by lymph node dissection of ND1a-ND2a-2. Table 1 summarizes the tumor characteristics. Histopathological examination revealed 145 (66.5%) adenocarcinomas, 53 (24.3%) squamous cell carcinomas, and 20 (9.2%) carcinomas of other NSCLC subtypes, including adenosquamous carcinoma, pleomorphic carcinoma, large cell carcinoma, large cell neuroendocrine carcinoma, carcinoid tumor, lymphoepithelioma-like carcinoma, and mucoepidermoid carcinoma. The median pathological tumor size (pT) was 22.0 (15.8–30.0) mm; the median SUVmax_e_ and SUVmax_d_ of the primary tumors were 3.3 (1.73–6.58) and 4.2 (2.0–8.68), respectively, whereas the median RI was 0.25 (0.08–0.37).

### Clinicopathological differences between the cohorts according to lymph node status

The clinicopathological characteristics of the patients were compared based on the presence or absence of lymph node metastases (Table 2). In total, 44 patients were pathological lymph node (pN) positive (+), while 174 patients were pN negative (0).

**Table 2:** Clinicopathological characteristics based on pathological N factor

Characteristics	Overall (*n* = 218)	pN 0 (*n* = 174)	pN 1 or 2 (*n* = 44)	*p* value
Sex				
Male	140	108	32	0.22*
Female	78	66	12	
Lobe				
RU	67	56	11	0.17*
RM	13	9	4	
RL	51	45	6	
LU	41	31	10	
LL	46	33	13	
Histological type				
Adenocarcinoma	145	116	29	0.43*
Squamous cell	53	44	9	
Other	20	14	6	
Clinical N stage				
cN0	187	160	27	< 0.001*
cN1or2	31	14	17	
Age, years	69.6 ± 9.5	69.5 ± 9.7	70.0 ± 8.5	0.73**
SUVmax_e_	3.3 [1.73–6.58]	2.9 [1.40–5.78]	5.7 [3.6–7.68]	< 0.001***
SUVmax_d_	4.2 [2.0– 8.68]	3.5 [1.45–7.70]	7.4 [4.35–10.58]	< 0.001***
RI	0.25 [0.08–0.37]	0.24 [0.01–0.36]	0.30 [0.23–0.39]	0.01***
pT, mm	22.0 [15.8–30.0]	21.0 [15.0–29.0]	25.5 [22.0–38.8]	< 0.001***

Data are presented as n (%), mean ± standard deviation, or median [interquartile ranges]

^*^Fisher's exact test; **unpaired *t* test; ***Mann–Whitney *U* test

*pN* pathological lymph node metastasis, *RU* right upper lung lobe, *RL* right lower lung lobe, *RM* right middle lung lobe, *LU* left upper lung lobe, *LL* left lower lung lobe, *SUVmax*_*e*_ early maximum standardized uptake value, *SUVmax*_*d*_ delayed maximum standardized uptake value, *RI* retention index, *pT* pathological tumor size

**Fig. 1:**
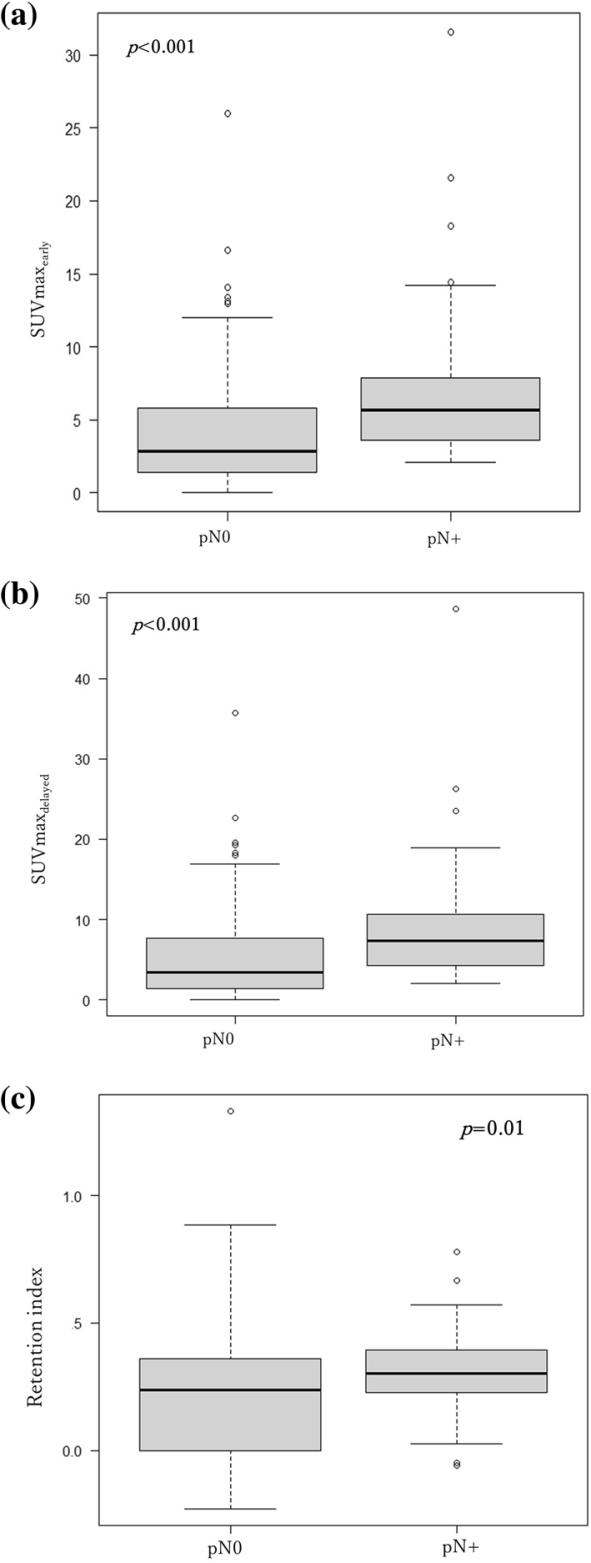
The **A** early maximum standardized uptake value (SUVmax_e_), **B** delayed maximum standardized uptake value (SUVmax_d_), and **C** retention index values were compared based on the presence of lymph node metastasis

Lymph node metastasis was confirmed in 44 tumors. The median RI was significantly higher in the pN(+) (*n* = 44) group (0.30, 0.23–0.39) compared to that in the N0 (*n* = 174) group (0.24, 0.01–0.36; *p* = 0.01) (Fig. 1).

**Fig. 2:**
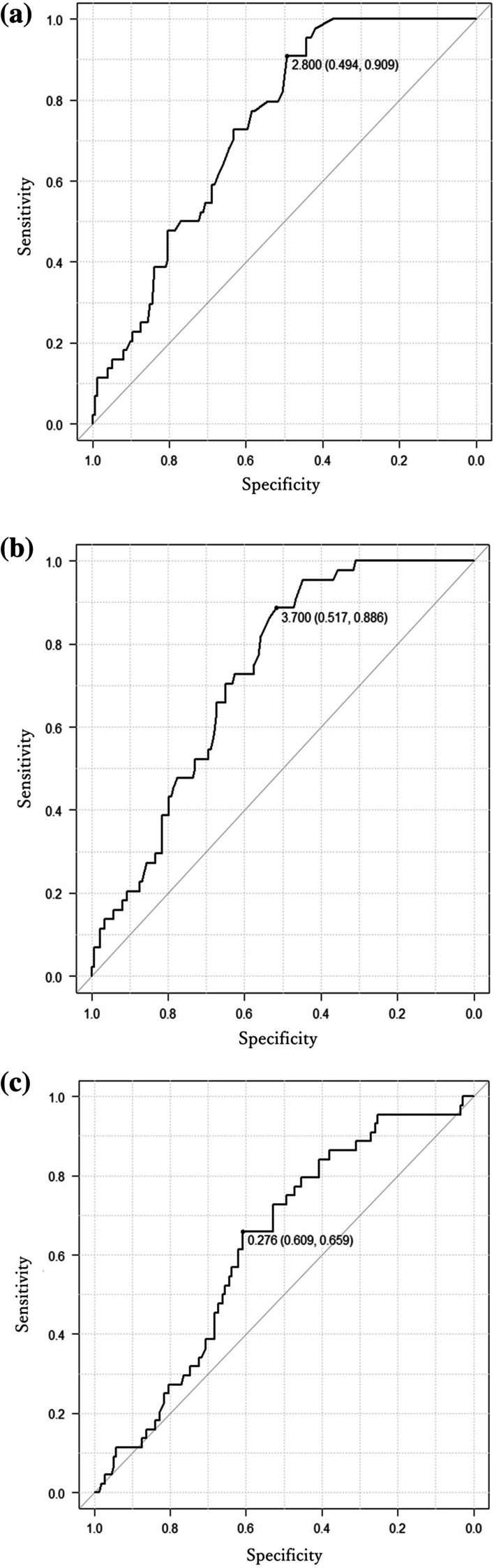
**A** Receiver operating characteristic (ROC) curve for early maximum standardized uptake value (SUVmax_e_) as a predictor of lymph node metastasis. The area under the ROC curve (AUC) was 0.73 with an SUVmax_e_ of 2.80 as the threshold; the sensitivity and specificity were 0.494 and 0.909, respectively. **B** ROC curve for delayed maximum standardized uptake value (SUVmax_d_) as a predictor of lymph node metastasis. The AUC was 0.73, with an SUVmax_d_ of 3.70 as the threshold, and the sensitivity and specificity were 0.517 and 0.886, respectively. **C** ROC curve for the retention index (RI) as a predictor of lymph node metastasis. The AUC of the RI was 0.62 (95% CI 0.54–0.71), and the threshold of the RI was 0.276; the sensitivity and specificity were 0.609 and 0.659, respectively

The area under the ROC curve (AUC) values for SUVmax_e_ and SUVmax_d_ as diagnostic markers of nodal metastasis were 0.73 (95% confidence intervals [CIs], 0.66–0.80) and 0.73 (95% CI 0.66–0.78), respectively. The SUVmax_e_ and SUVmax_d_ thresholds were 2.80 and 3.70, respectively. The AUC for RI in the diagnosis of nodal metastasis was 0.62 (95% CI 0.54–0.71), and the RI threshold was 0.276 (Fig. 2).

### Histopathological analysis

In patients with adenocarcinoma (*n* = 145), the median RI values of the pN0 (*n* = 116) and pN(+) (*n* = 29) groups were 0.16 (0.0–0.30) and 0.29 (0.17–0.40), respectively. The RI of the primary tumor was significantly higher in the pN+ group compared to the pN0 group (*p* < 0.01). In patients with squamous cell carcinoma (*n* = 53), median RI values did not differ significantly between the pN0 group (0.33, IQR 0.25–0.45) and the pN(+) group (0.33, IQR 0.28–0.44) (*p* = 0.62). In patients with other NSCLC subtypes (*n* = 20), there was also no significant difference in the median RI between the pN0 group (0.23, IQR 0.17–0.38) and the pN(+) group (0.29, IQR 0.26–0.30) (*p* = 0.34) (Table 3).

**Table 3:** Clinicopathological characteristics based on pathological tumor stage and histopathological subtype

Histopathological subtype	Value	pN0	pN(+)	*p* value
Adenocarcinoma (*n* = 145)		(*n* = 116)	(*n* = 29)	
	SUVmax_e_	2.1 [1.08–3.38]	4.3 [3.0–7.2]	< 0.001
	SUVmax_d_	2.6 [1.2–4.7]	5.9 [4.1–8.9]	< 0.001
	RI	0.16 [0.0–0.30]	0.29 [0.17–0.40]	< 0.01
	pT	19 [14–25]	25 [21–30]	< 0.01
Squamous cell carcinoma (*n* = 53)		(*n* = 44)	(*n* = 9)	
	SUVmax_e_	5.9 [3.77–8.55]	9.3 [6.60–14.20]	0.02
	SUVmax_d_	8.1 [4.58–11.95]	13.4 [9.4–18.5]	0.03
	RI	0.33 [0.25–0.45]	0.33 [0.28–0.44]	0.62
	pT	27 [19.8–32.0]	33 [32–42]	0.05
Other (*n* = 20)		(*n* = 14)	(*n* = 6)	
	SUVmax_e_	5.3 [2.28–8.28]	7.0 [6.65–7.43]	0.39
	SUVmax_d_	6.5 [2.88–11.10]	9.1 [8.53–9.75]	0.39
	RI	0.23 [0.17–0.38]	0.29 [0.26–0.30]	0.34
	pT	21.5 [12.3–27.0]	27.0 [24.8–44.3]	0.23

*RI* retention index, *SUVmax*_*e*_ early maximum standardized uptake value, *SUVmax*_*d*_ delayed maximum standardized uptake value, *pT* pathological tumor size

**Fig. 3:**
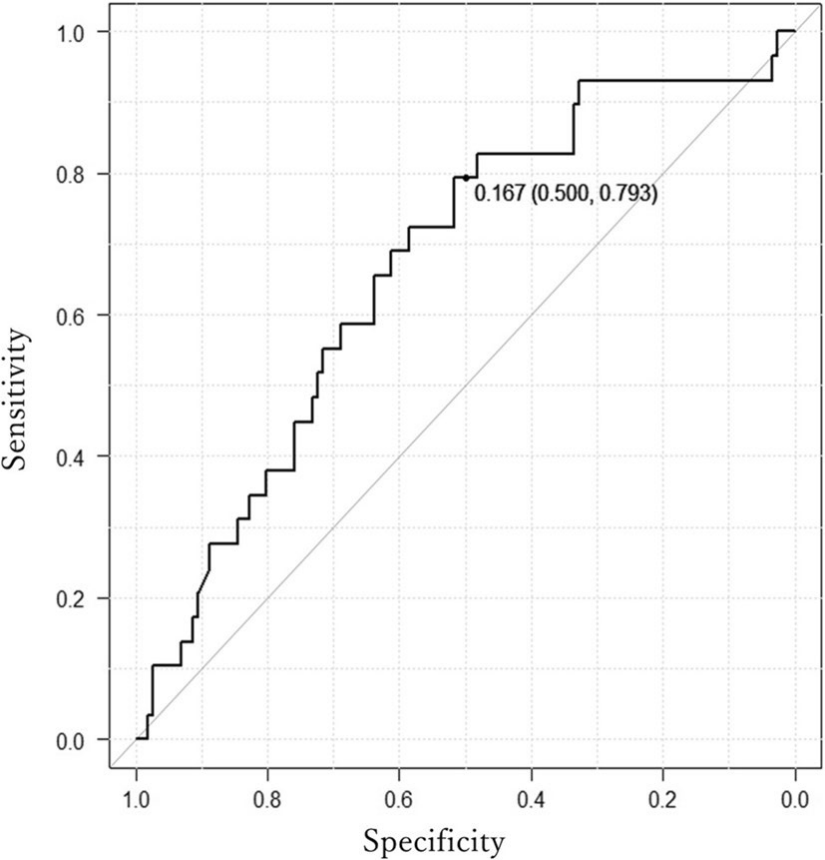
Receiver operating characteristic (ROC) curve for the retention index (RI) in the adenocarcinoma for lymph node metastasis. The area under the ROC curve (AUC) of the RI was 0.67 (95% CI 0.56–0.78), the threshold of RI for the nodal metastasis was 0.167, and the sensitivity and specificity were 0.500 and 0.793, respectively

**Fig. 4:**
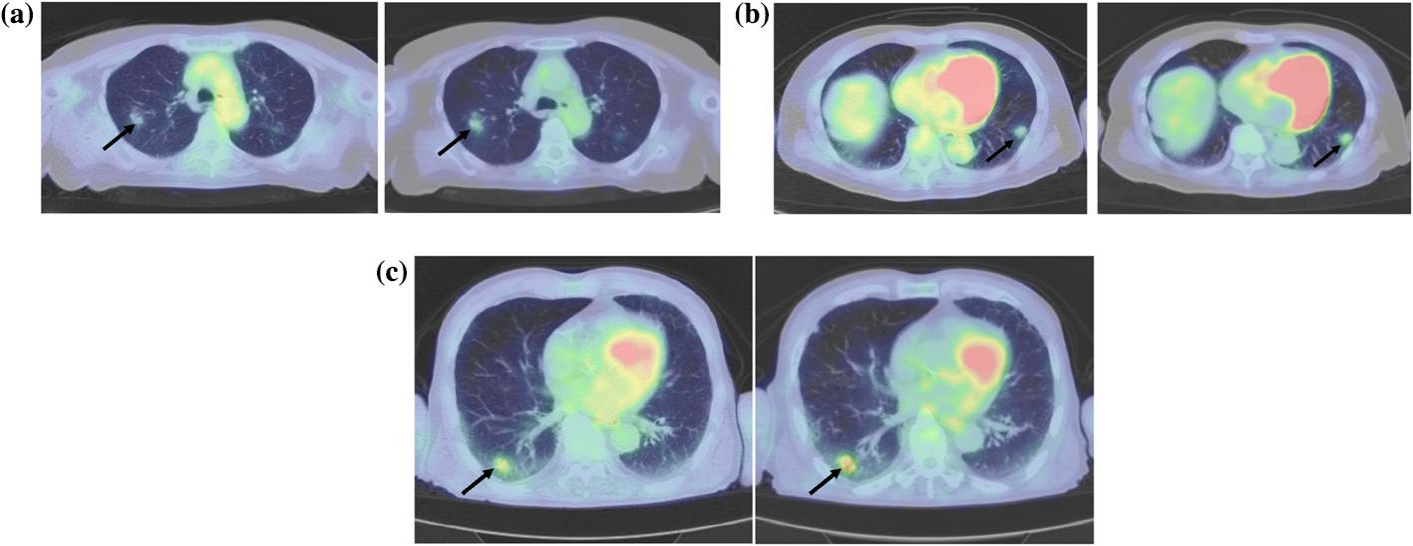
**A** Adenocarcinoma with no lymph node metastasis. The RI of the primary tumor (arrow) was low at 0.15 (left: SUVmax_e_ 1.3, right: SUVmax_d_ 1.5). **B** Adenocarcinoma with lymph node metastasis. The primary tumor (arrow) exhibited a high RI of 0.53 (left: SUVmax_e_ 1.7, right: SUVmax_d_ 2.6). **C** Squamous cell carcinoma with no lymph node metastasis. The primary tumor (arrow) showed a moderate RI of 0.39 (left: SUVmax_e_ 2.3, right: SUVmax_d_ 3.2)

Figure 3 shows the ROC curves for RI as a diagnostic marker of nodal metastasis in patients with adenocarcinoma. The AUC for lymph node metastasis diagnosis in adenocarcinoma was 0.67 (95% CI 0.56–0.78), and the threshold of the RI was 0.167; sensitivity was 0.500, and specificity was 0.793 (Fig. 3). Table 4 shows a comparison of RI values between different histological subtypes. RI values were significantly higher in squamous cell carcinoma than those in adenocarcinoma. Figure 4 shows three representative tumor samples with different RI characteristics.

**Table 4:**
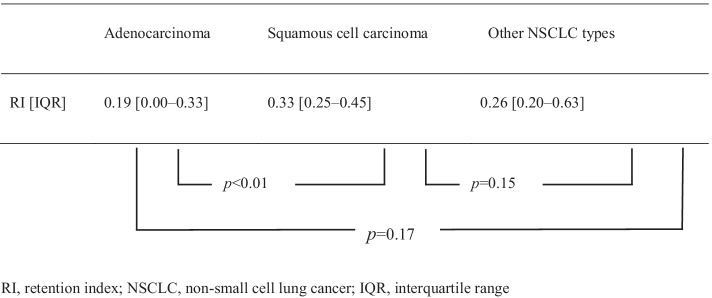
Comparison of retention index values by cell carcinoma histological type

*RI* retention index, *NSCLC* non-small cell lung cancer, *IQR* interquartile range

### Correlation between tumor size and retention index

**Fig. 5:**
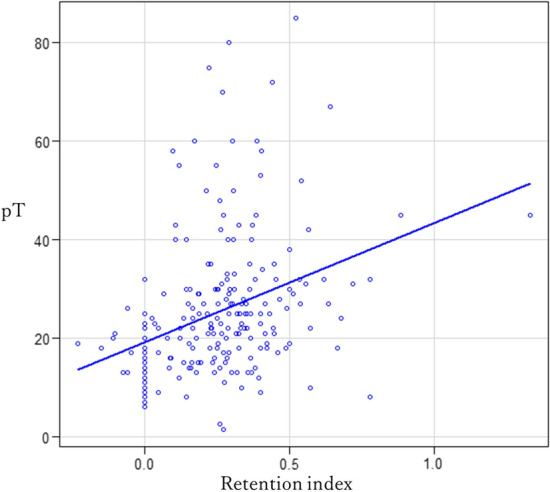
Correlation coefficient value between the retention index and pathological tumor size in all the histological types was 0.35 (95% CI 0.23–0.46; *p* < 0.01)

We further investigated the correlation between tumor size and RI since the results (Table 3) implied that in all histological types, the tumor size and RI had a positive correlation. The correlation coefficient value between the RI and pT in all histological types was 0.35 (95% CI 0.23–0.46, *p* < 0.01) (Fig. 5). Based on the histological type, in the adenocarcinoma group (*n* = 145), the correlation between the primary tumor size and the RI was 0.34 (95% CI 0.18–0.47, *p* < 0.01); in the squamous cell carcinoma group (*n* = 53), the correlation was 0.21 (95% CI − 0.07 to 0.45, *p* = 0.14); and for the other histological subtypes, the correlation was 0.63 (95% CI 0.23–0.83, *p* < 0.01) (Table 5). In the squamous cell carcinoma group (*n* = 53), there was no positive correlation between tumor size and RI.

**Table 5:** Correlation coefficient values between RI and pT by histological type

Histological type	Correlation coefficient	95% CI	*p* value
All (*n* = 218)	0.35	0.23–0.46	< 0.01
Adenocarcinoma (*n* = 145)	0.34	0.18–0.47	< 0.01
Squamous cell carcinoma (*n* = 53)	0.21	− 0.07 to 0.45	0.14
Other (*n* = 20)	0.63	0.23–0.83	< 0.01

*CI* confidence interval, *RI* retention index, *pT* pathological tumor size

### Multivariable analysis

We used a multivariable logistic regression to adjust for the potentially confounding roles of RI and tumor size on lymph node metastasis (Table 6). In a multivariable analysis, primary tumor RI was not an independent risk factor for lymph node metastasis (odds ratio [OR]: 2.53, 95% CI 0.47–13.50, *p* = 0.28) in all histopathological subtypes. However, in the adenocarcinoma group, a higher RI value of the primary tumor was associated with an increased risk for lymph node metastasis (OR: 12.3, 95% CI 1.11–135.0, *p* < 0.05).

**Table 6:** Multivariable analyses of predictors of lymph node metastasis in patients

Histological type	Variable	OR	95% CI	*p* value
All (*n* = 218)	pT	1.03	1.01–1.05	0.01
	RI	2.53	0.47–13.50	0.28
Adenocarcinoma (*n* = 145)	pT	1.02	1.00–1.05	0.11
	RI	12.3	1.11–135.0	0.04
Squamous cell carcinoma (*n* = 53)	pT	1.07	1.01–1.13	0.03
	RI	0.8	0.04–17.6	0.89
Other (*n* = 20)	pT	1.03	0.96–1.11	0.38
	RI	0.34	0.00–988.0	0.79

*OR* odds ratio, *CI* confidence interval, *RI* retention index, *pT* pathological tumor size

## Discussion

In this study, we examined the implications of the RI of the primary tumor as a predictor of lymph node metastasis in patients NSCLC. Several studies have examined the role of tumor SUVmax as a risk marker for nodal metastasis (Takahashi et al. 2019; Noda et al. 2016; Nakamura et al. 2015; Karam et al. 2018). The RI of the lymph node is also a predictor of lymph node metastasis in NSCLC, and a higher RI of the primary tumor is known to predict risk for distant metastasis and poor recurrence-free survival (Shinya et al. 2009, 2019; Kim et al. 2012; Satoh et al. 2012; Shimizu et al. 2015). However, the role of the RI of the primary tumor as a predictor of lymph node metastasis in NSCLC remains unclear. To the best of our knowledge, no studies have been conducted in an attempt to compare the RI of the primary tumor as a predictor of lymph node metastasis between different histological subtypes of NSCLC.

We demonstrated that, in patients with adenocarcinoma, the RI of the primary tumor was a significant predictor of lymph node metastasis; however, in patients with squamous cell carcinoma, and those with other NSCLC types, the RI of the primary tumor was not a significant predictor of lymph node metastasis.

RI values were higher in patients with squamous cell carcinoma than in those with adenocarcinoma. However, the RI was a reliable predictor of lymph node metastasis only in the adenocarcinoma group. We also demonstrated a correlation between the RI value and tumor size across all NSCLC types. Multivariable analysis demonstrated that based on histology, the RI of the primary tumor was an independent risk factor for lymph node metastasis only in the adenocarcinoma group. The RI of the primary tumor was therefore a reliable predictor of lymph node metastasis in patients with adenocarcinoma.

Similar histopathological tendencies of the FDG-PET/CT SUVmax have been reported in other studies. The single-time-point FDG-PET/CT SUVmax of the primary tumor was significantly higher in squamous cell carcinoma than in adenocarcinoma. However, the significance of the SUVmax as a prognostic factor was stronger in adenocarcinoma and weaker in squamous epithelial carcinoma (Tsutani et al. 2011; Wang et al. 2015). Glucose metabolism abnormalities might explain the differences in the role of the SUVmax between adenocarcinoma and squamous cell carcinoma (Tsutani et al. 2011; Wang et al. 2015). Tumor glucose transporter (GLUT)-1 overexpression is associated with high FDG uptake. GLUT-1 is fully (100%) expressed in squamous cell carcinoma, but only partially (58%) expressed in adenocarcinoma (Tsutani et al. 2011; Wang et al. 2015). GLUT-1 expression could explain the variable significance of SUVs as a predictor of lymph node metastasis in different histological subtypes evident in our study. This biological difference likely modified the effect of the RI as a risk factor among different histopathological subtypes.

We consider that, in general, the SUVmax and related indices (including the RI) are sensitive indicators of malignant potential in patients with adenocarcinoma.

Till date, no study has investigated in detail the clinical features and SUVmax or RI of patients in the other NSCLC groups. Furthermore, since the number of patients with other NSCLC subtypes was small, we could not determine the clinical behavior of those subgroups.

Numerous studies have mentioned that a high SUVmax of the primary tumor is a risk factor for lymph node metastasis, and the cutoff SUVmax of the primary tumor ranges from 2.5 to 4.0 (Takahashi et al. 2019; Nakamura et al. 2015; Karam et al. 2018; Li et al. 2013; Kanzaki et al. 2011; Nakahashi et al. 2020; Zhai et al. 2020; Kaseda et al. 2016). SUVmax data at the 60-min time-point following F18-FDG administration in many studies of single-time-point 18F-PET-CT examination are similar to the SUVmax_e_ demonstrated in this study, showing that an SUVmax_e_ cutoff of 2.8 was a predictor of nodal metastasis. However, SUVmax values vary between facilities, and variations of up to 30% across three institutions have been reported (Westerterp et al. 2007). We consider the RI using DTP PET-CT a reliable assessment tool that does not require standardization since it is the ratio of the two SUVmax values. The RI is a simple value that can be widely used in many facilities with DTP PET-CT scanning data. Additionally, in the real world, the SUVmax of hilum and mediastinum lymph nodes is often not calculated.

We consider that the primary tumor’s RI could help identify nodal metastasis in adenocarcinoma in real-world clinical practice.

This study had some limitations, including a retrospective, single-center design. Excluding the AIS, MIA, and old BAC categories from pathologic diagnoses constituted another limitation. This decision was motivated by our focus on targeting clear invasive adenocarcinoma. We thus did not formally consider the differences between the Union for International Cancer Control TNM 7th and 8th edition guidelines on adenocarcinoma in our study. Furthermore, the number of cases of other NSCLC types was small, which may have affected our results.

## Conclusions

This study demonstrated that the RI of the primary tumor on DTP FDG-PET imaging could be a useful predictor of lymph node metastasis in patients with adenocarcinoma compared to other cancer subtypes.

## Data Availability

The datasets used and analyzed during this study are available from the corresponding author upon reasonable request.

## Abbreviations

18F-FDG: 18F-fluorodeoxyglucose
AIS: Adenocarcinoma in situ
BAC: Bronchioloalveolar carcinoma
CI: Confidence index
CT: Computed tomography
DTP: Dual time-point
MIA: Minimally invasive adenocarcinoma
NSCLC: Non small-cell lung cancer
OR: Odds ratio
PET: Positron emission tomography
pT: Pathological tumor size
RI: Retention index
ROC: Receiver operating curve
SUVmax: Maximum standardized uptake value
